# Soluble Neuropilin-1 is an independent marker of poor prognosis in early breast cancer

**DOI:** 10.1007/s00432-021-03635-1

**Published:** 2021-04-21

**Authors:** Tilman D. Rachner, Sabine Kasimir-Bauer, Andy Goebel, Kati Erdmann, Oliver Hoffmann, Martina Rauner, Lorenz C. Hofbauer, Rainer Kimmig, Ann-Kathrin Bittner

**Affiliations:** 1 Division of Endocrinology and Metabolic Bone Diseases, Diabetes and Bone Diseases, Department of Medicine III, TU Dresden, Fetscherstraße 74, 01307 Dresden, Germany; 2 Center for Healthy Ageing, Department of Medicine III, TU Dresden, Dresden, Germany; 3 Department of Gynecology and Obstetrics, University Hospital Essen, University of Duisburg-Essen, Essen, Germany; 4 German Cancer Consortium (DKTK), Dresden and German Cancer Research Center (DKFZ), Heidelberg, Germany; 5 Department of Urology, TU Dresden, Dresden, Germany; 6 National Center for Tumor Diseases (NCT), Dresden, Germany

**Keywords:** Neuropilin-1, Breast cancer, Prognosis

## Abstract

**Background:**

Neuropilin-1 (NRP-1) is a transmembrane protein that acts as a multifunctional non-tyrosine kinase receptor with an established role in development and immunity. NRP-1 also regulates tumor biology, and high expression levels of tissue NRP-1 have been associated with a poor prognosis. Recently, ELISA-based quantification of soluble NRP-1 (sNRP-1) has become available, but little is known about the prognostic value of sNRP-1 in malignancies.

**Materials and methods:**

We measured sNRP-1 in the serum of 509 patients with primary early breast cancer (BC) at the time of diagnosis using ELISA.

**Results:**

Mean serum values of sNRP-1 were 1.88 ± 0.52 nmol/l (= 130.83 ± 36.24 ng/ml). SNRP-1 levels weakly correlated with age, and were higher in peri- and postmenopausal patients compared to premenopausal patients, respectively (*p* < 0.0001). Low levels of sNRP-1 were associated with a significant survival benefit compared to high sNRP-1 levels at baseline (*p* = 0.005; HR 1.94; 95%CI 1.23–3.06). These findings remained significant after adjustment for tumor stage including lymph node involvement, grading, hormone receptor, HER2 status, and age (*p* = 0.022; HR 1.78; 95%CI 1.09–2.91).

**Conclusion:**

Our findings warrant further investigations into the prognostic and therapeutic potential of sNRP-1 in BC.

## Introduction

Breast cancer (BC) is the most common cancer in women worldwide. Despite major improvements in diagnosis, treatment and a 5-year overall survival (OS) rate of 87%, approximately 8% of the patients develop distant metastasis within 5 years after primary diagnosis, showing a great variety depending on the genetic background, tumor stage, lymph node involvement as well as proliferation index of the primary tumor (Ki67) (Hölzel et al. 2017). Currently, only a few prognostic and therapeutic targets have been identified and further markers are needed to identify patients at high risk of recurrence and to define new specific targets for affected patients (Perez-Gracia et al. 2017).

Neuropilin-1 (NRP-1), also known as CD304 or BDCA-4, is a transmembrane protein and a multifunctional non-tyrosine kinase receptor that plays an established role in development and immunity (Pellet-Many et al. 2008; Romeo et al. 2002). NRP-1 is expressed by endothelial cells as well as other cell types and was first identified as a receptor for class 3 semaphorins (SEMA3) (Kolodkin et al. 1997). It is assumed that NRP-1 promotes cancer by binding to molecules involved in angiogenesis and epithelial-to-mesenchymal Transition (EMT) (Soker et al. 1998; Chu et al. 2014). It is known to act as a co-receptor for a vascular endothelial growth factor (VEGF), placenta growth factor (PlGF), transforming growth factor β1 (TGF-β1), hepatocyte growth factor (HGF), platelet-derived growth factor (PDGF) and fibroblast growth factors (FGFs) among others (Mamluk et al. 2002; Glinka and Prudhomme 2008; Hu et al. 2007; Cao et al. 2010). The interaction of NRP-1 with VEGF and VEGFR2 has been of special interest since it results in an enhanced signaling and promotion of angiogenesis. In the past years, NRP-1 has also been associated with malignant transformation and cancer progression (reviewed in (Prudhomme and Glinka 2012)).

NRP-1 is also expressed in BC cells and has been associated with effects on cell invasion, survival, and migration (Bachelder et al. 2002) and negatively correlates with patient survival (Ghosh et al. 2008; Jubb et al. 2012). Furthermore, NRP-1 expression in BC tissue has been linked to lymph node metastasis (Seifi-Alan et al. 2018). This data is consistent with further studies, showing a correlation of NRP-1 expression in tumor tissue and aggressiveness of BC (Luo et al. (2016)).

Soluble NRP-1 (sNRP-1) can be successfully quantified using ELISA-based approaches (Lu et al. 2009) and more recently, plasma and tumor tissue levels of NRP-1 were found to be upregulated in node-positive patients with BC and advanced metastatic disease (Naik et al. 2017). However, little is known about the prognostic value of serum NRP-1 levels in affected patients. This study aimed to assess sNRP-1 levels in serum samples of women with early BC at the time of the first diagnosis and to investigate whether these findings are associated with the prognosis and clinical parameters.

## Patients and methods

### Patient population and study design

This retrospectively assessed study was conducted in a well-established cohort of 509 early BC patients as previously described (Rachner et al. 2018, 2020). Briefly, patients were diagnosed between 2004 und 2009 and serum samples were obtained prior to any systemic or surgical therapy after written informed consent using a protocol approved by the clinical Ethics committee of the University Hospital Essen (05/2856). To be eligible for study inclusion, patients were required to have histologically proven BC, no severe uncontrolled co-morbidities or medical conditions, no further present malignancies or malignancies in the past. Treatment was performed according to the current guidelines at the time including adjuvant chemotherapy (anthracyclines, 5-fluorouracil, taxanes, cyclophosphamide), anti-hormonal therapy in the case of hormone-responsive tumors (tamoxifen or an aromatase inhibitor), trastuzumab in the case of HER2-positivity (following FDA approval in 2006) and radiotherapy. Patients receiving neo-adjuvant chemotherapy were not included. Tumor type, TNM-staging and grading were assessed at the Institute of Pathology of the University Hospital Essen as part of the West German Comprehensive Cancer Center.

### Sampling of serum

Blood was collected with an S-Monovette (Sarstedt AG & Co.) from each patient, stored at 4 °C and processed within 4 h to avoid blood cell lysis. Blood fractionation was carried out by centrifugation for 10 min at 2500×*g*. Subsequently, 3–4 ml of the upper phase, constituting blood serum, were removed and frozen at − 80 °C (as previously described (Rachner et al. 2018)).

### Detection of sNRP-1 by ELISA

Assessment of all serum samples was conducted at the same time. SNRP-1 was detected by ELISA (Biomedica, Vienna, Austria). With a sensitivity of 0.09 nmol/l (= 6.3 ng/ml) and an in-between and within-run precision of < 12% CV this 1 day, 4 h assay detects free and ligand-bound sNRP-1.

Briefly, 10 µl of the sample was used per well and the ELISA was conducted according to the manufacturer’s protocol. After pipetting the standards, controls and samples into the respective wells of the pre-dilution plate, 10 µl of guanidine hydrochloride was added for 30 min and 200 µl of assay buffer was added. In the pre-coated plate, 50 µl of assay buffer was added and 50 µl of the samples from the pre-dilution plate was transferred. Following this step, 50 µl of biotinylated anti-NRP-1 antibody was added to each well and plates were incubated for 2 h at room temperature. Following a number of washing steps, 150 µl of conjugate was added to each well and plates were incubated for another hour. Following another set of washing steps, 150 µl of substrate was given into each well and after 30 min of incubation, 50 µl of stop solution was applied. Absorbance was measured immediately at 450 nm with reference at 630 nm using FLUOstar Omega (BMG Labtech, Ortenberg, Germany).

### Statistical analysis

Results are presented as the standard deviation of the mean unless otherwise stated. Groups of two were assessed by the Mann–Whitney-*U*-Test, groups of three or more were assessed by ANOVA. Correlation analysis was performed using the Pearson correlation coefficient.

Samples were divided into two groups at the median sNRP-1 value and classified as sNRP-1^high^ and sNRP-1^low^. Kaplan–Meier curves were assessed using the log-rank (Mantel-Cox) test. BC-specific survival (BCSS) was defined as time between diagnosis of the primary tumor and death directly related to the disease. Multivariate Cox regression analyses were performed to identify prognostic factors for the different survival endpoints. The multivariate Cox’s regression models were adjusted to known clinical prognostic factors in BC patients. *p* values < 0.05 were considered statistically significant.

## Results

From the 509 patients included in this cohort, 506 samples were available for assessment and valid sNRP-1 measurements were obtained in all cases (506/506). Mean serum values of sNRP-1 were 1.88 ± 0.52 nmol/l (= 130.83 ± 36.24 ng/ml). There was no significant difference in samples when stratifying according to tumor size (T) or lymph node involvement (N). Furthermore, the estrogen receptor (ER) status of the tumor did not influence sNRP-1 levels).

**Fig. 1:**
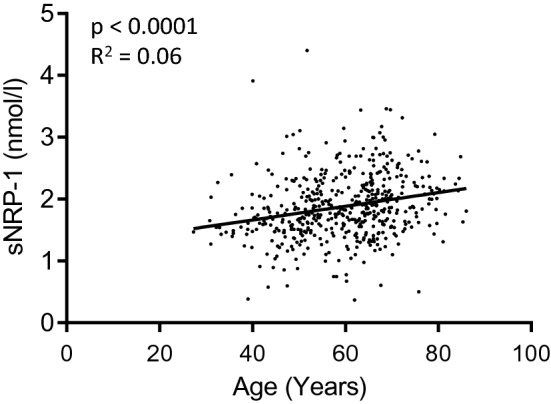
sNRP-1 levels weakly correlate with age. sNRP-1 serum levels positively correlate with the age of assessed subjects

SNRP-1 levels were significantly higher in the serum of patients who were older than 60 years of age at the time of diagnosis, compared to younger patients (1.96 ± 0.51 vs. 1.79 ± 0.52; *p* < 0.0001). In line with this finding, sNRP-1 levels were significantly higher in peri- and postmenopausal patients compared to premenopausal patients, respectively (Table 1). There was no significant difference between peri- and postmenopausal samples. Age and sNRP-1 levels were weakly positively correlated (*p* < 0.0001, *r* 0.24, *r*^2^ 0.06) (Fig. 1).

**Table 1:** Soluble NRP-1 levels in breast cancer patients

	Patients (*n*)	sNRP-1 (nmol/l)	*p* value
Age (years)			
< 60	243	1.79 ± 0.52	0.0001
> 60	263	1.96 ± 0.51	
Menopausal status			
Premenopausal	72	1.65 ± 0.50	< 0.0001 vs. post- 0.04 vs. post-
Perimenopausal	63	1.77 ± 0.48	
Postmenopausal	371	1.94 ± 0.52	
Histology			
Ductal	383	1.89 ± 0.52	ns
Lobular	68	1.88 ± 0.54	
Others	55	1.84 ± 0.47	
Tumor size			
pT1	320	1.87 ± 0.54	ns
pT2	158	1.88 ± 0.50	
pT3-4	24	2.03 ± 0.46	
Nodal status			
Node negative	338	1.86 ± 0.52	ns
Node positive	158	1.92 ± 0.52	
Grading			
I	88	1.88 ± 0.47	ns
II	269	1.87 ± 0.51	
III	148	1.89 ± 0.57	
ER status			
Negative	94	1.91 ± 0.66	ns
Positive	411	1.87 ± 0.48	
PR status			
Negative	135	1.88 ± 0.62	ns
Positive	370	1.88 ± 0.48	
Her2 status			
Negative	423	1.88 ± 0.50	ns
Positive	80	1.86 ± 0.64	

Values of sNRP-1 are given in nmol/l. *ns* not significant; Differences to total number of patients (*n* = 506) may occur, since for some characteristics information may be missing

### Soluble NRP-1 is an independent prognostic marker for BC specific survival

**Fig. 2:**
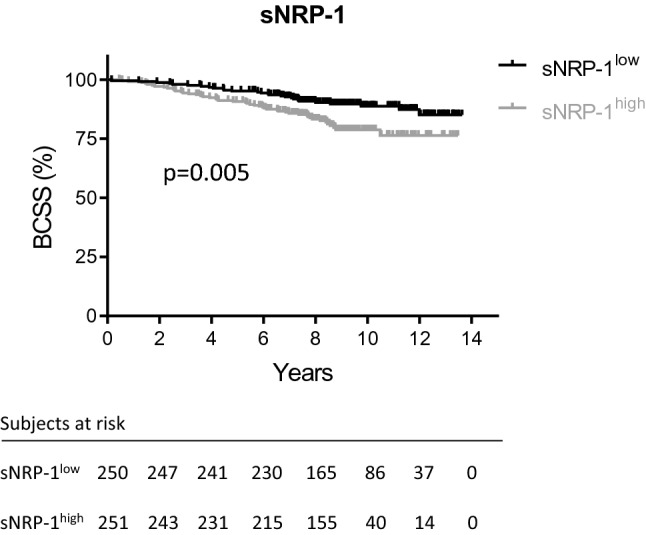
sNRP-1 is a prognostic marker for breast cancer-specific survival. Baseline levels of sNRP-1 were measured in 506 patients diagnosed with primary, non-metastatic breast cancer using ELISA. The patient cohort was divided at the median into a sNRP-1^high^ and sNRP-1^low^ group. Kaplan–Meier curve for breast cancer-specific survival (BCSS) was assessed using the log-rank (Mantel-Cox) test based on survival data of 501 patients

Follow-up was available for 501 of the 506 patients, with five patients lost to follow-up. Mean follow-up was 8.6 years (range 0.2–13.6 years) and 74 cases of BC-specific deaths were documented during that time. When stratifying patients according to their sNRP-1 levels into a sNRP-1^high^ and sNRP-1^low^ group, there was a significant survival benefit for patients who had low sNRP-1 levels at baseline (*p* = 0.005; HR 1.94; 95%CI 1.23–3.06). While there were 47 documented cases of BC-specific death in the sNRP-1^high^ group, only 27 cases were seen in the sNRP-1^low^ cohort despite a slightly longer mean follow-up (8.08 vs. 9.04 years, respectively) (Fig. 2).

Multivariate Cox regression analyses adjusted for tumor stage including lymph node involvement, grading, hormone receptor and HER2 status revealed sNRP-1 to be an independent prognostic marker for BCSS (*p* = 0.006; HR 1.98; 95%CI 1.22–3.21).

**Fig. 3:**
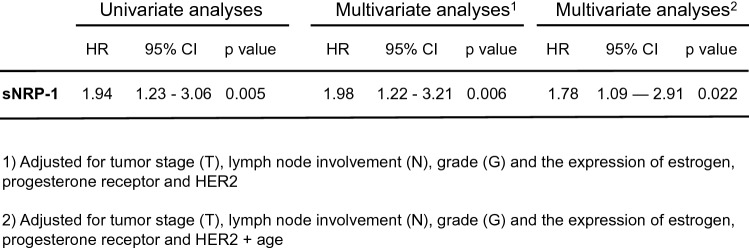
sNRP-1 is an independent prognostic marker in breast cancer. Univariate and multivariate Cox regression analyses for BCSS dependent on sNRP-1 levels and clinicopathological parameters were performed. Multivariate analyses adjusted for tumor stage (T), lymph node involvement (N), grade (G) and the expression of estrogen, progesterone receptor and HER2 as well as age confirmed the independent prognostic value of sNRP-1 in breast cancer. All included parameters were used in a binominal fashion (sNRP-1: high vs low; tumor stage: pT4, pT3, pT2 vs pT1; lymph node involvement: positive vs negative; grade: III, II vs I; hormone receptor and HER2 status: positive vs negative; age: > 60 vs < 60)

Since we saw a positive correlation between age and sNRP-1 levels, we also included age in the multivariate Cox regression analyses in addition to the factors mentioned above. Here, sNRP-1 remained an independent prognostic marker for BCSS (*p* = 0.022; HR 1.78; 95%CI 1.09–2.91) (Fig. 3). Results also remained significant when menopausal status was included in addition to age and the other parameters (*p* = 0.015; HR 1.86; 95%CI 1.13–3.07).

## Discussion

In the past years, the role of NRP-1 in cancer progression has gained significant interest with studies showing a tumor promoting or prognostic role in several malignancies including pancreas (Ben et al. 2014), liver (Xu and Xia 2013), gastric (Kang et al. 2020), colon (Parikh et al. 2004), lung (Roche et al. 2002) and breast cancer (Ferrario et al. 2006). Consequently, NRP-1 is considered a potential target for cancer therapy (Ding et al. 2018). More precisely, as an enhancer of VEGF activity and mediator of endothelial cell adhesion to extracellular matrix proteins, NRP-1 has been considered as a potential antiangiogenic target.

Angiogenesis is an essential step to provide the basis for tumor progression at a local and metastatic level. In pancreatic ductal adenocarcinoma, microvessel density was significantly higher in the tumors with high NRP-1 expression than that in the tumors with low NRP-1 expression (Ben et al. 2014).

In prostate cancer, shRNA-mediated suppression of NRP-1 regulated the invasiveness and metastatic dissemination of cancer cells in vivo and NRP-1 expression was established as an independent marker of metastasis and cancer-specific survival (Tse et al. 2017). Using a Neuropilin-1 transmembrane domain interfering peptide, the proliferation of BC cell lines was inhibited and tumor size was reduced in murine models of breast cancer (Arpel et al. 2016). Furthermore, a humanized monoclonal antibody against NRP-1 is currently being investigated in clinical trials (NCT00747734, NCT00954642).

As an additional approach, the use of sNRP-1 as a decoy receptor has been investigated in animal models of tumor growth. SNRP-1 inhibited tumor angiogenesis and growth in murine granulocytic sarcoma (chloroma) (Gagnon et al. 2000) and significantly prolonged survival in a murine model of systemic leukemia (Schuch et al. 2002). However, the majority of studies has investigated tissue expression of NRP-1 rather than its soluble form. The perception that sNRP-1 has opposing effects to membrane bound NRP-1 (Uniewicz et al. 2011) would imply that high levels of sNRP-1 would favor a better prognosis in malignancies. However, there is currently very limited data assessing the prognostic value of sNRP-1. In a study in early cervical cancer and cervical intraepithelial neoplasia conducted in a small cohort of 56 patients, sNRP-1 correlated with stage and more importantly showed a significant correlation with tissue expression (Yang et al. 2015). More recently, circulating and tumor tissue expression of NRP-1 were found to increase in advanced nodal and metastatic BC (Naik et al. 2017). These findings suggest that high levels of circulating sNRP-1 may in fact be a reflection of its level of tissue expression. This would imply that measurement of circulating sNRP-1 may be a more convenient way to determine NRP-1 activity than the more invasive way of analyzing tissue. Recent data also suggest that changes of sNRP-1 following the initiation of an anticancer-therapy may be useful for monitoring treatment response. In patients receiving neo-adjuvant chemotherapy for locally advanced BC, significantly increased plasma sNRP-1 levels were observed in patients with a pathological partial response (*p* = 0.018) (Al-Zeheimi et al. 2019).

This study lacks the possibility to analyze corresponding tissue expression of NRP-1 in the breast tumors. However, in this large and well-characterized cohort, the measurement of sNRP-1 was shown to be an independent prognostic marker for cancer survival in BC and further studies are warranted to increase our knowledge of how sNRP-1 may be utilized as a prognostic and/or therapeutic target in BC.
